# Pregnancy and risk of COVID‐19: a Norwegian registry‐linkage study

**DOI:** 10.1111/1471-0528.16969

**Published:** 2021-11-01

**Authors:** MC Magnus, L Oakley, HK Gjessing, O Stephansson, HM Engjom, F Macsali, PB Juliusson, A‐M Nybo Andersen, SE Håberg

**Affiliations:** ^1^ Centre for Fertility and Health Norwegian Institute of Public Health Oslo Norway; ^2^ MRC Integrative Epidemiology Unit at the University of Bristol Bristol UK; ^3^ Department of Non‐communicable Disease Epidemiology London School of Hygiene and Tropical Medicine London UK; ^4^ Department of Global Public Health and Primary Care University of Bergen Bergen Norway; ^5^ Division of Clinical Epidemiology Department of Medicine, Solna Karolinska Institutet Stockholm Sweden; ^6^ Department of Women's Health Karolinska University Hospital, Solna Stockholm Sweden; ^7^ Department of Health Registry Research and Development Norwegian Institute of Public Health Oslo Norway; ^8^ Department of Obstetrics and Gynecology Haukeland University Hospital Bergen Norway; ^9^ Department of Clinical Science University of Bergen Bergen Norway; ^10^ Department of Public Health University of Copenhagen Copenhagen Denmark

**Keywords:** COVID‐19, pregnancy, SARS‐CoV‐2

## Abstract

**Objective:**

To compare the risk of acute respiratory syndrome coronavirus 2 (SARS‐CoV‐2) infection and contact with specialist healthcare services for coronavirus disease 2019 (COVID‐19) between pregnant and non‐pregnant women.

**Population or sample:**

All women ages 15–45 living in Norway on 1 March 2020 (*n* = 1 033 699).

**Methods:**

We linked information from the national birth, patient, communicable diseases and education databases using unique national identifiers.

**Main outcome measure:**

We estimated hazard ratios (HR) among pregnant compared to non‐pregnant women of having a positive test for SARS‐CoV‐2, a diagnosis of COVID‐19 in specialist healthcare, or hospitalisation with COVID‐19 using Cox regression. Multivariable analyses adjusted for age, marital status, education, income, country of birth and underlying medical conditions.

**Results:**

Pregnant women were not more likely to be tested for or to a have a positive SARS‐CoV‐2 test (adjusted HR 0.99; 95% CI 0.92–1.07). Pregnant women had higher risk of hospitalisation with COVID‐19 (HR 4.70, 95% CI 3.51–6.30) and any type of specialist care for COVID‐19 (HR 3.46, 95% CI 2.89–4.14). Pregnant women born outside Scandinavia were less likely to be tested, and at higher risk of a positive test (HR 2.37, 95% CI 2.51–8.87). Compared with pregnant Scandinavian‐born women, pregnant women with minority background had a higher risk of hospitalisation with COVID‐19 (HR 4.72, 95% CI 2.51–8.87).

**Conclusion:**

Pregnant women were not more likely to be infected with SARS‐CoV‐2. Still, pregnant women with COVID‐19, especially those born outside of Scandinavia, were more likely to be hospitalised.

**Tweetable abstract:**

Pregnant women are at increased risk of hospitalisation for COVID‐19.

## Tweetable abstract

Pregnant women are at increased risk of hospitalisation for COVID‐19.

## Introduction

It is unclear whether pregnant women have an increased risk of severe acute respiratory syndrome coronavirus 2 (SARS‐CoV‐2) infection, but emerging evidence suggests that pregnant women may have a higher risk of severe coronavirus disease 2019 (COVID‐19) if infected.^1^, ^2^, ^3^, ^4^ However, the evidence is not consistent.^5^ Most existing studies were from single centres or on hospitalised women with COVID‐19, and investigated whether pregnancy increased the risk of severe disease, admission to intensive‐care units, mechanical ventilation and death.^6^, ^7^ Population‐based estimates comparing pregnant women with non‐pregnant women are lacking.

The aim of this study was to compare the risk of acute respiratory syndrome coronavirus 2 (SARS‐CoV‐2) infection and contact with specialist healthcare services for coronavirus disease 2019 (COVID‐19) between pregnant and non‐pregnant women. We used data from national health registries on all women in Norway between 15 and 45 years of age. Notably, Norway has not conducted universal testing of pregnant or delivering women.

## Methods

### Study population and data sources

We followed all women between 15 and 45 years of age registered in the Norwegian National Population Registry on 1 March 2020 (*n* = 1 033 699), until 28 February 2021. Information on pregnancies and antenatal care visits was obtained from the birth registry, the patient registry (covering specialist/secondary healthcare services) and the general practitioner database (covering general practitioners/primary healthcare services).^8^ Information on SARS‐CoV‐2 tests was provided by the Norwegian Surveillance System for Communicable Diseases, and contacts with specialist healthcare services for suspected and confirmed COVID‐19 were obtained from the patient registry. Information on education (highest level attained as of 2019) and household income (in 2018) was from Statistics Norway. Data was linked by using unique personal identification numbers. Data from all registries was provided by the Emergency Preparedness Register for COVID‐19 at the Norwegian Institute of Public Health.^9^ More information on data sources is available in the Supporting Information. Norwegian legislation does not require consent from individuals to conduct research using the national health registries. Ethical approval was obtained for this study from the Regional Committee of Medical and Health Research Ethics of South/East Norway (reference number 141135).

### Definition of completed pregnancies

The birth registry provided data on live births, stillbirths, fetal losses and induced abortions after 12 gestational weeks. Registrations of miscarriages and induced abortions occurring before 12 gestational weeks were obtained from the patient registry and the general practitioner database, as previously described.^10^ The diagnostic codes used to define miscarriage and induced abortion are shown in Table S1. These early miscarriages and induced abortions do not have registration information on gestational length of the pregnancy. Based on the mean gestational length for all induced abortions in Norway in the anonymous abortion registry, and the gestational age distribution of miscarriages from the literature,^11^ we assigned these pregnancies a gestational duration of 8 weeks, and in sensitivity analyses a gestational duration of 6 or 10 weeks.

### Definition of ongoing pregnancies

We identified ongoing pregnancies using codes for antenatal care visits in the general practitioner database and the patient registry (Table S2). These antenatal codes capture virtually all pregnancies that eventually will be recorded in the birth registry, as 99.5% of pregnancies in the birth registry had at least one registration of these codes during pregnancy. For a pregnancy to be defined as ‘ongoing’ at the end of the study period, we excluded registrations occurring within the duration of a completed pregnancy. Second, we required that registrations of the antenatal codes were at least 90 days after a completed pregnancy to be counted as a new/currently ongoing pregnancy. Antenatal codes are not registered with a gestational length. Based on the distribution of the first registration of an antenatal code for the already completed pregnancies in the birth registry (Figure S1), we defined the start date of ongoing pregnancies to be 5 weeks (35 days) before the first antenatal consultation, assuming that very few women have an antenatal visit before 5 weeks of pregnancy. In additional analyses we assigned these pregnancies to start 10 weeks before the first visit.

### COVID‐19

We defined COVID‐19 in three ways: (1) a positive test for SARS‐CoV‐2, (2) any diagnosis of COVID‐19 in specialist healthcare, and (3) hospitalisation with confirmed COVID‐19. Two new ICD‐10 codes were implemented at the start of the pandemic: U07.1 ‘COVID‐19 with confirmed virus’; and U07.2 ‘COVID‐19 without confirmed virus’. Notably, registration of confirmed COVID‐19 (U07.1) requires a positive test for SARS‐CoV‐2. We used both codes to define specialist‐diagnosed COVID‐19. We assumed that these women had symptoms of COVID‐19 which warranted contact with specialist healthcare services. We further analysed hospitalisation for confirmed COVID‐19 (U07.1) separately.

### Pre‐existing chronic conditions

We obtained information on a wide range of pre‐existing chronic condition defined as risk factors for severe COVID‐19.^12^ The diagnostic codes we used to define these conditions are shown in Table S3. We required at least two registrations from January 2017 until end of follow‐up to qualify as an existing underlying condition.

### Statistical analysis

We used Cox proportional hazards models on calendar time to examine separately whether pregnant women had an increased risk of (1) a positive test; (2) a specialist care diagnosis of COVID‐19; and (3) hospitalisation with confirmed COVID‐19. Women were followed from 1 March 2020, until the event of interest; emigration, death or reaching 28 February 2021 without an event was treated as censoring. Pregnancy status was a time‐varying exposure, allowing women to contribute both pregnant and non‐pregnant follow‐up time. We used robust cluster variance estimation with the woman’s identification number as the cluster variable. We estimated unadjusted associations, and associations with adjustment for marital status (single, married/cohabitating or other), educational level (elementary school, high‐school, vocational, up to 4 years of higher education, and more than 4 years of higher education), household income (categorised into tertiles), country of birth (Scandinavian countries [Norway, Sweden and Denmark] or non‐Scandinavian countries), and chronic conditions. We first analysed the entire follow‐up period and subsequently analysed the two main waves of the pandemic in Norway separately (1 March to 30 June 2020, and 1 July 2020 to 28 February 2021).^13^ We also evaluated whether associations differed with pregnancy trimester (1st trimester: ≤83 days; 2nd trimester: 84–195 days; 3rd trimester: ≥196 days). As a higher risk of COVID‐19 has been reported among non‐Scandinavian ethnic groups in Norway,^14^ we also examined the risk of COVID‐19 separately for Scandinavian‐ and non‐Scandinavian‐born women.

It could be that pregnant women were tested more often, and that milder COVID‐19 therefore was detected more often among pregnant women, resulting in higher estimates of COVID‐19 among pregnant women. We examined whether pregnant women were tested more often than non‐pregnant women. Women could have multiple tests during follow‐up. We used the Andersen and Gill recurrent events Cox model,^15^ where women continued to be a part of the risk set until emigration, death or end of follow‐up. To evaluate whether testing in relation to admission to hospital for delivery or miscarriage/abortion was driving the associations, we performed sub‐analyses where we excluded tests conducted within 3 days before or after a pregnancy ended, and in addition hospitalisations where the end of pregnancy was within a hospital stay for COVID‐19. All analyses were conducted in STATA version 16 (StataCorp, College Station, TX, USA).

### Patient and public involvement

No patients were involved directly in the design of the study, recruitment, or conduct of the study because our cohort consisted of normal individuals from the population at large (not patients).

## Results

Of the 1 033 699 women included in the study, 101 820 (10%) had been pregnant during the follow‐up time. There were 35 915 (4%) who were still pregnant at the end of follow‐up (ongoing pregnancies). There was a slightly higher proportion of women born outside of Scandinavia among the pregnant women than among non‐pregnant women (Table 1). Fewer pregnant women had chronic underlying risk conditions (Table 1).

**Table 1:** Distribution of characteristics among 1 033 699 women aged 15–45 in Norway who were pregnant between 1 March 2020 and 28 February 2021

Characteristics	Women who were pregnant (*n* = 102 820)	Women who were not pregnant (*n* = 930 879)
Age at start of follow‐up, mean (SD)	30.8 (5.1)	30.2 (8.8)
Country of birth, *n* (%)		
Norway	73 936 (71.9)	705 553 (75.8)
Another Scandinavian country	2 026 (2.0)	14 186 (1.5)
Outside of Scandinavia	26 528 (25.8)	208 193 (22.4)
Unknown	330 (0.3)	2 947 (0.3)
Marital status, *n* (%)		
Single	59 163 (57.5)	636 473 (68.4)
Married/registered partner	39 520 (38.4)	241 090 (25.9)
Other	4 137 (4.0)	53 316 (5.7)
Educational level, *n* (%)		
Elementary school	16 243 (15.8)	221 684 (23.8)
Highschool	19 416 (18.9)	230 053 (24.7)
Vocational	1 566 (1.5)	13 955 (1.5)
Up to 4 years of university	37 289 (36.3)	272 101 (29.2)
More than 4 years of university	20 049 (19.5)	102 543 (11.0)
Unknown	8 257 (8.0)	90 543 (9.7)
Household income, *n* (%)		
1st tertile (≤500 730 NOK)	30 241 (29.4)	304 914 (32.8)
2nd tertile (500 731–846 668 NOK)	41 219 (40.1)	293 937 (31.6)
3rd tertile (>846 668 NOK)	28 081 (27.3)	307 073 (33.0)
Unknown	3 279 (3.2)	24 955 (2.7)
Chronic conditions, *n* (%)		
Diabetes	1 203 (1.2)	10 365 (1.1)
Cerebrovascular disease	104 (0.1)	1 339 (0.1)
Other chronic cardiovascular disorders	823 (0.8)	6 783 (0.7)
Immune deficiency	37 (0.04)	453 (0.05)
Reduced immune function due to medications	1 566 (1.5)	14 713 (1.6)
Chronic lung disease	3 505 (3.4)	36 953 (4.0)
Neurological disorders	93 (0.1)	2 263 (0.2)
Kidney failure	27 (0.03)	507 (0.05)
Organ transplant	21 (0.02)	628 (0.07)
Haematological cancer	95 (0.1)	1 036 (0.1)
Other types of cancer	94 (0.1)	2 405 (0.3)

### Risk of a positive SARS‐CoV‐2 test

The overall rate of a positive SARS‐CoV‐2 test among women aged 15–45 years was 5 per 100 000 person‐days. The risk of a positive test was similar for pregnant women and non‐pregnant women (adjusted HR 0.99, 95% CI 0.92–1.07), with similar HRs across all trimesters (Table 2). The estimate was similar for the two waves of the pandemic (first wave, adjusted HR 0.94, 95% CI 0.76–1.17, and second wave, adjusted HR 1.00, 95% CI 0.92–1.08; Table 2). Results were also similar after excluding women with positive tests within 3 days around the end of pregnancy (Table S4). Women born outside of Scandinavia had an increased risk of a positive test compared with Scandinavian women in general, and an even higher risk when pregnant (adjusted HR 2.37, 95% CI 1.98–2.84) when compared with pregnant Scandinavian women (Table S5).

**Table 2:** Hazard ratio of a positive SARS‐CoV‐2 test during pregnancy among 1 033 698^a^ women in Norway between 15 and 45 years of age

Follow‐up period	Pregnancy status	Follow‐up time in days	No. of positive tests	Hazard ratio (95% CI)	
				Unadjusted	Adjusted^b^
Complete follow‐up^c^	Non‐pregnant	356,383 248	16 364	1.00	1.00
	Pregnant	15 481 516	708	0.98 (0.91–1.05)	0.99 (0.92–1.07)
	1st trimester	5 454 096	256	0.97 (0.86–1.10)	0.98 (0.87–1.11)
	2nd trimester	5 787 833	271	0.99 (0.88–1.12)	1.01 (0.90–1.14)
	3rd trimester	4 239 587	181	0.96 (0.83–1.11)	0.97 (0.84–1.13)
Wave 1^d^	Non‐pregnant	119 435 417	1977	1.00	1.00
	Pregnant	5 198 569	87	1.01 (0.82–1.26)	0.94 (0.76–1.17)
	1st trimester	1 746 753	24	0.87 (0.58–1.30)	0.81 (0.54–1.21)
	2nd trimester	1 941 362	35	1.05 (0.75–1.46)	0.97 (0.69–1.36)
	3rd trimester	1 510 454	28	1.13 (0.78–1.65)	1.05 (0.72–1.53)
Wave 2^e^	Non‐pregnant	236 947 831	14 387	1.00	1.00
	Pregnant	10 282 947	621	0.97 (0.90–1.05)	1.00 (0.92–1.08)
	1st trimester	3 707 343	232	0.98 (0.86–1.12)	1.01 (0.88–1.15)
	2nd trimester	3 846 471	236	0.99 (0.87–1.12)	1.02 (0.90–1.16)
	3rd trimester	2 729 133	153	0.93 (0.79–1.09)	0.96 (0.82–1.12)

^a^ Excluded one person who tested positive before 1 March 2020.

^b^ Adjusted for age as a linear and squared term, country of birth, marital status, education, household income, diabetes, cerebrovascular disease, other cardiovascular disorders, immune deficiency, chronic lung disease, reduced immune function, neurological disorders, kidney failure, organ transplant, haematological cancer, and other types of cancer.

^c^ 1 March 2020–28 February 2021.

^d^ 1 March 2020–30 June 2020.

^e^ 1 July 2020–28 February 2021.

### Risk of specialist‐care diagnosis and hospitalisation

The overall rate of a specialist healthcare diagnosis of COVID‐19 was 0.3 per 100 000 person days, while the rate of being hospitalised with confirmed COVID‐19 was 0.1 per 100 000 person days. Pregnant women had an increased risk of a specialist‐care diagnosis of COVID‐19 (adjusted HR 3.46, 95% CI 2.89–4.14), which was similar in both waves of the pandemic (Table 3). The risk appeared to be highest in the third trimester but was attenuated when we excluded pregnancies ending within the same hospital stay as for COVID‐19 (Table 3). The increased risk of contact with specialist healthcare services for COVID‐19 while pregnant were higher in non‐Scandinavian pregnant women (adjusted HR 7.50, 95% CI 5.76–9.77) and in Scandinavian pregnant women (adjusted HR 2.66, 95% CI 2.09–3.39) when compared with Scandinavian women who were not pregnant (Table S6).

**Table 3:** Hazard ratio of a COVID‐19 diagnosis in specialist healthcare services for pregnant women among 1 033 696^a^ women between 15 and 45 years of age in Norway

Follow‐up period	Pregnancy status	Follow‐up time in days	All events			Excluding events where the end of pregnancy occurred within the hospital stay for COVID‐19	
			No. of events	Hazard ratio (95% CI)		No. of events	Hazard ratio (95% CI)
				Unadjusted	Adjusted^b^		Adjusted^b^
Complete follow‐up^c^	Non‐pregnant	358 063 481	900	1.00	1.00	900	1.00
	Pregnant	15 549 308	144	3.66 (3.07–4.36)	3.46 (2.89–4.14)	87	2.11 (1.68–2.64)
	1st trimester	5 479 349	36	2.63 (1.89–3.68)	2.48 (1.77–3.47)	24	1.67 (1.11–2.51)
	2nd trimester	5 813 675	28	1.86 (1.28–2.71)	1.76 (1.20–2.57)	27	1.71 (1.16–2.51)
	3rd trimester	4 256 284	80	7.53 (6.00–9.47)	7.16 (5.68–9.01)	36	3.25 (2.33–4.54)
Wave 1^d^	Non‐pregnant	119 573 874	291	1.00	1.00	291	1.00
	Pregnant	5 203 614	50	3.96 (2.93–5.34)	3.32 (2.42–4.54)	29	1.91 (1.29–2.82)
	1st trimester	1 748 663	12	2.93 (1.65–5.21)	2.49 (1.39–4.46)	7	1.44 (0.68–3.07)
	2nd trimester	1 943 283	7	1.43 (0.68–3.03)	1.20 (0.56–2.55)	7	1.19 (0.56–2.52)
	3rd trimester	1 511 668	31	8.50 (5.87–12.30)	7.06 (4.81–10.35)	15	3.38 (1.99–5.72)
Wave 2^e^	Non‐pregnant	238 489 607	609	1.00	1.00	609	1.00
	Pregnant	10 345 694	94	3.52 (2.83–4.37)	3.53 (2.83–4.40)	58	2.21 (1.69–2.91)
	1st trimester	3 730 686	24	2.51 (1.67–3.77)	2.50 (1.65–3.78)	17	1.80 (1.11–2.92)
	2nd trimester	3 870 392	21	2.06 (1.34–3.19)	2.08 (1.34–3.23)	20	2.01 (1.28–3.15)
	3rd trimester	2 744 616	49	7.03 (5.26–9.41)	7.09 (5.30–9.47)	21	3.09 (2.00–4.76)

^a^ Excluded three people in contact with specialist healthcare services for suspected or confirmed COVID‐19 disease before 1 March 2020.

^b^ Adjusted for age as a linear and squared term, country of birth, marital status, education, household income, diabetes, cerebrovascular disease, other cardiovascular disorders, immune deficiency, chronic lung disease, reduced immune function, neurological disorders, kidney failure, organ transplant, haematological cancer and other types of cancer.

^c^ 1 March 2020–28 February 2021.

^d^ 1 March 2020–30 June 2020.

^e^ 1 July 2020–28 February 2021.

Pregnant women had a substantially higher risk of being hospitalised for confirmed COVID‐19 (adjusted HR 4.70, 95% CI 3.51–6.30) in both waves of the pandemic (Table 4). The greatest risk was seen in the third trimester, though the trimester‐specific differences were attenuated when we excluded pregnancies ending within the same hospital stay where COVID‐19 was diagnosed. Among COVID‐19 hospitalised women, the proportion who also had diagnoses of lower respiratory illness (ICD‐10 codes J12–J22, J80, J96) was 32% in pregnant and 49% in non‐pregnant women. The median number of days in hospital was 2 for pregnant (mean 3.3 days) and 2 for non‐pregnant women (mean 3.7 days).

**Table 4:** Hazard ratio of hospitalisation (event) with confirmed COVID‐19 for pregnant women among 1 033 699 women between 15 and 45 years of age

Follow‐up period	Pregnancy status	Follow‐up time in days	All events			Excluding events where the end of pregnancy occurred within the hospital stay for COVID‐19	
			No. of events	Hazard ratio (95% CI)		No. of events	Hazard ratio (95% CI)
				Unadjusted	Adjusted*		Adjusted*
Complete follow‐up**	Non‐pregnant	358 173 181	289	1.00	1.00	289	1.00
	Pregnant	15 559 886	53	4.19 (3.12–5.61)	4.70 (3.51–6.30)	24	2.21 (1.45–3.37)
	1st trimester	5 482 901	8	1.81 (0.89–3.66)	2.00 (0.99–4.06)	6	1.55 (0.69–3.49)
	2nd trimester	5 817 698	11	2.27 (1.25–4.15)	2.58 (1.41–4.72)	10	2.44 (1.30–4.59)
	3rd trimester	4 259 287	34	10.01 (7.01–14.27)	11.37 (7.97–16.21)	8	2.78 (1.37–5.65)
Wave 1***	Non‐pregnant	119 591 018	88	1.00	1.00	88	1.00
	Pregnant	5 205 118	15	3.93 (2.27–6.80)	4.17 (2.37–7.31)	6	1.70 (0.73–3.97)
Wave 2****	Non‐pregnant	238 582 163	201	1.00	1.00	201	1.00
	Pregnant	10 354 768	38	4.30 (3.04–6.08)	4.96 (3.52–6.98)	18	2.45 (1.51–3.98)

* Adjusted for age as a linear and squared term, country of birth, marital status, education, household income, diabetes, cerebrovascular disease, other cardiovascular disorders, immune deficiency, chronic lung disease, reduced immune function, neurological disorders, kidney failure, organ transplant, haematological cancer, and other types of cancer.

** 1 March 2020–28 February 2021.

*** 1 March 2020–30 June 2020.

**** 1 July 2020–28 February 2021.

Both being pregnant and being non‐Scandinavian increased the risk of hospitalisation with confirmed COVID‐19, and pregnant non‐Scandinavian women were at highest risk of hospitalisation with COVID‐19 (Table S7).

### Likelihood of being tested for SARS‐CoV‐2

The SARS‐CoV‐2 testing rate was 310 tests per 100 000 person days. Overall, pregnant women were slightly less likely to be tested for SARS‐CoV‐2 (adjusted HR 0.90, 95% CI 0.88–0.91) (Table S8). The rate of testing in pregnant versus non‐pregnant women has been similar or lower after the initial pandemic months (Figure S2). Lowest test rates among pregnant women were seen during third trimester (Table S8). Non‐Scandinavian women had lower probability of testing, especially when pregnant (adjusted HR 0.72, 95% CI 0.70–0.74) compared with non‐pregnant Scandinavian women (Table S9).

In additional analyses we reassigned the gestational duration of pregnancies ending in miscarriages and induced abortions to be 6 and 10 weeks, and ongoing pregnancies to start 10 weeks prior to the first antenatal visit instead of 5 weeks; the results were very similar to the main analyses.

## Discussion

### Main findings

We found no overall increased risk of a positive SARS‐CoV‐2 test among pregnant women compared with non‐pregnant women. However, pregnant women were at a substantially increased risk of receiving specialist healthcare and also hospitalisation. Women born outside of Scandinavia were less likely to be tested and were at a particularly higher risk of being hospitalised for COVID‐19 when pregnant compared with Scandinavian‐born women.

### Strengths and limitations

This study is unique in its size as it included all women of reproductive age in Norway, with the ability to compare the pregnant with the non‐pregnant population of similar age. We were also able to examine whether differences in testing behaviour were likely to influence results, which was not found to be the case.

A limitation of registry studies is that health definitions rely on registrations from contact with healthcare. Norway has not conducted universal testing of pregnant or delivering women. Testing was therefore by indication on either having symptoms of COVID‐19, due to workplace testing or having been exposed to someone who has tested positive for SARS‐CoV‐2. Asymptomatic individuals, or those with very mild symptoms, were unlikely to get tested. Test capacity for SARS‐CoV‐2 and healthcare availability for those with milder COVID‐19 symptoms have also varied throughout the pandemic. In the initial phase, testing was limited, and testing for Covid‐19 was prioritised to those with severe symptoms or underlying risk conditions. Our results indicated that pregnant women were slightly more likely to be tested in the initial phase than were non‐pregnant women, but after the initial months when testing capacity increased, pregnant women were slightly less likely to be tested. Still, results stratified according to the two main waves of the pandemic in Norway yielded similar estimates, supporting that test availability was unlikely to explain our findings. The association with being tested while pregnant may not be generalisable to other countries with different testing strategies. We were not able to evaluate other measures of severity such admission to intensive care unit due to small numbers (15 events in the age group of interest).

Identifying ongoing pregnancies and early terminations through healthcare contacts is also prone to misclassification. Towards the end of the follow‐up period we were less likely to capture ongoing pregnancies that will end in miscarriage or induced abortions. Only 44.2% of miscarriages and induced abortions had a prior antenatal code. This could have resulted in underestimation of the number of pregnant women and attenuation of associations. As antenatal visits do not provide information on gestational length, we defined pregnancy start date and durations for ongoing pregnancies and early abortions based on known distributions. We chose a strict approach in the main analyses to minimise misclassification of ‘non‐pregnant’ days as ‘pregnant’, which likely resulted in some true ‘pregnant’ days being counted as ‘non‐pregnant’ days. However, several sensitivity analyses with other assumptions of gestational lengths for these pregnancies yielded very similar results, indicating little impact on associations.

Another limitation was that we could not adjust for some potential confounding factors, such as crowded living conditions, body mass index or smoking. We were not able to look at other measures of severity such as admission to intensive care units due to small numbers. Even though we were able to study all women of reproductive age in Norway, our findings might not be generalisable outside of Scandinavia or other European countries with universal healthcare coverage.

### Interpretation

Women born outside of Scandinavia were less likely to be tested and were at a particularly higher risk of being hospitalised for COVID‐19 when pregnant compared with Scandinavian‐born women. An increased risk of COVID‐19 among ethnic minorities has been reported in several countries,^16^, ^17^ including Norway.^13^ This has been attributed to crowded households and more service‐related professions with personal contact. We observed less testing among both pregnant and non‐pregnant women born outside of Scandinavia. A higher threshold for testing may have resulted in more severe illness before seeking healthcare, which is supported by our findings of increased risk of specialist care and hospitalisations than Scandinavian‐born women. Routine testing of minority women in connection with antenatal care could reduce these differences.

In line with some previous studies,^1^, ^4^, ^6^ although not all,^5^ our results support that pregnant women may experience more severe symptoms as part of COVID‐19; however, our results may also reflect a lower threshold for hospitalisation of pregnant women with COVID‐19 than for non‐pregnant women. In our study, we could only look at hospitalisation as a marker of severity. Notably, prior studies did not compare pregnant and non‐pregnant women in the general population. Among hospitalised women, others have found that pregnant women have an increased risk of intensive care and death when compared with non‐pregnant women.^1^, ^6^ A recent meta‐analysis of 123 176 non‐pregnant and 10 000 pregnant women reported a higher case‐fatality rate in pregnant women.^7^ As pregnant women may be more likely to be admitted to hospitals than non‐pregnant women with similar symptoms, restricting studies to women hospitalised with COVID‐19 may complicate interpretation of results. We found a higher risk of hospitalisation when pregnant, but a similar duration of the hospital stays and slightly lower proportion with co‐registrations of lower respiratory illness, compared with non‐pregnant women. This may suggest that, in Norway, when hospitalised, there is no substantial difference in severity of disease in pregnant women, although more detailed data are needed to address this.

Even though several studies have concluded that pregnant women are at higher risk of severe COVID‐19,^2^ and of adverse pregnancy outcomes in women with COVID‐19,^6^, ^18^ vaccination of pregnant women against COVID‐19 is currently debated.^19^, ^20^, ^21^, ^22^ COVID‐19 vaccines have not been tested in pregnant women, and pregnant women are in general not recommended vaccination, although this may be evaluated on an individual basis.^23^, ^24^ We found that pregnant women were not at higher risk of SARS‐CoV2 infection *per se*, however, our results support the current evidence that there may be an increased risk of hospitalisation when infected during pregnancy. Protecting pregnant women against COVID‐19 is therefore important, and there is an urgent need to address vaccine safety in pregnancy.

## Conclusions

In this large nationwide registry study, pregnant women were not at higher risk of SARS‐CoV‐2 infection, but pregnancy increased the risk of receiving specialist care and hospitalisation for COVID‐19 compared with non‐pregnant women of the same age. Pregnant women born outside of Scandinavia were at particularly increased risk, and increased surveillance in this group is warranted. The increased risk of hospitalisation for COVID‐19 supports the need for vaccination of pregnant women.

### Disclosure of interests

All authors report no conflict of interest.

### Contribution to authorship

All authors contributed to the study design, acquisition, analyses and interpretation of the data. MCM drafted the initial manuscript and LO, HKG, OS, HME, FM, PBJ, AMNA and SEH critically revised the manuscript for important intellectual content. Final approval of the version to be published was given by all authors. The corresponding author attests that all listed authors meet the authorship criteria and that no others meeting the criteria have been omitted.

### Details of ethics approval

This research was approved by the Regional Committee of Medical and Health Research Ethics of South/East Norway (reference number 141135).

### Funding

This research was supported by the Research Council of Norway through its Centres of Excellence funding scheme (project number 262700). This work was (partly) funded by Nordforsk through the funding to SCOPE ‐ Scandinavian studies of COvid‐19 in PrEgnancy, project number 105545. MCM has received funding from the European Research Council (ERC) under the European Union’s Horizon 2020 research and innovation programme (grant agreement No. 947684). The funders had no role in the completion of the research project, the writing of the manuscript for publication or the decision to publish the results.

### Acknowledgements

None.

## Supporting information


**Figure S1.** Gestational age distribution of the first registration of antenatal codes.
**Figure S2.** Probability of testing in pregnant versus non‐pregnant women per month.
**Table S1.** Codes used to define miscarriages and induced abortions.
**Table S2.** Codes used to identify ongoing pregnancies.
**Table S3.** Codes used to define underlying conditions and risk groups.
**Table S4.** Risk of a positive COVID‐19 test during pregnancy, excluding those who were positive 3 days before or after the end of pregnancy.
**Table S5.** Risk of a positive COVID‐19 test in women born outside of Scandinavia.
**Table S6.** Risk of specialist COVID‐19 diagnoses in women born outside of Scandinavia.
**Table S7.** Risk of COVID‐19 hospitalisation in women born outside Scandinavia.
**Table S8.** The probability of being tested for COVID‐19 when pregnant.
**Table S9.** The probability of being tested for COVID‐19 when pregnant in women born outside Scandinavia.

Supplementary Material

Supplementary Material

Supplementary Material

Supplementary Material

Supplementary Material

Supplementary Material

Supplementary Material

Supplementary Material

Supplementary Material

## Data Availability

Data are available by applying to the Norwegian registry owners: https://helsedata.no/soknadsveiledning/. The data are not publicly available due to privacy and ethical restrictions.
